# WNT Signaling and Bone: Lessons From Skeletal Dysplasias and Disorders

**DOI:** 10.3389/fendo.2020.00165

**Published:** 2020-04-09

**Authors:** Yentl Huybrechts, Geert Mortier, Eveline Boudin, Wim Van Hul

**Affiliations:** Center of Medical Genetics, Antwerp University Hospital, University of Antwerp, Antwerp, Belgium

**Keywords:** skeletal dysplasias, Wingless and Int-1 (WNT)/planar cell polarity (PCP) pathway, Wingless and int-1 (WNT)/β-catenin pathway, Wingless and int-1 (WNT)/Ca^2+^ pathway, osteoporosis

## Abstract

Skeletal dysplasias are a diverse group of heritable diseases affecting bone and cartilage growth. Throughout the years, the molecular defect underlying many of the diseases has been identified. These identifications led to novel insights in the mechanisms regulating bone and cartilage growth and homeostasis. One of the pathways that is clearly important during skeletal development and bone homeostasis is the Wingless and int-1 (WNT) signaling pathway. So far, three different WNT signaling pathways have been described, which are all activated by binding of the WNT ligands to the Frizzled (FZD) receptors. In this review, we discuss the skeletal disorders that are included in the latest nosology of skeletal disorders and that are caused by genetic defects involving the WNT signaling pathway. The number of skeletal disorders caused by defects in WNT signaling genes and the clinical phenotype associated with these disorders illustrate the importance of the WNT signaling pathway during skeletal development as well as later on in life to maintain bone mass. The knowledge gained through the identification of the genes underlying these monogenic conditions is used for the identification of novel therapeutic targets. For example, the genes underlying disorders with altered bone mass are all involved in the canonical WNT signaling pathway. Consequently, targeting this pathway is one of the major strategies to increase bone mass in patients with osteoporosis. In addition to increasing the insights in the pathways regulating skeletal development and bone homeostasis, knowledge of rare skeletal dysplasias can also be used to predict possible adverse effects of these novel drug targets. Therefore, this review gives an overview of the skeletal and extra-skeletal phenotype of the different skeletal disorders linked to the WNT signaling pathway.

## Introduction

In the last decades, plenty of examples have been generated where the identification of causal genes for rare, monogenic diseases resulted in novel insights into the role and functioning of already known pathways. A textbook example of this is Wingless and int-1 (WNT; pronounced “wint”) signaling and its role in bone formation and homeostasis. First indications of this involvement were generated by studying rare monogenic conditions with abnormal bone mass, delivering novel targets for drug development for osteoporosis, a common disorder characterized by decreased bone mass in late life. Recently, an antibody against a WNT signaling inhibitor was approved as an anabolic agent for the treatment of osteoporosis.

**Figure 1:**
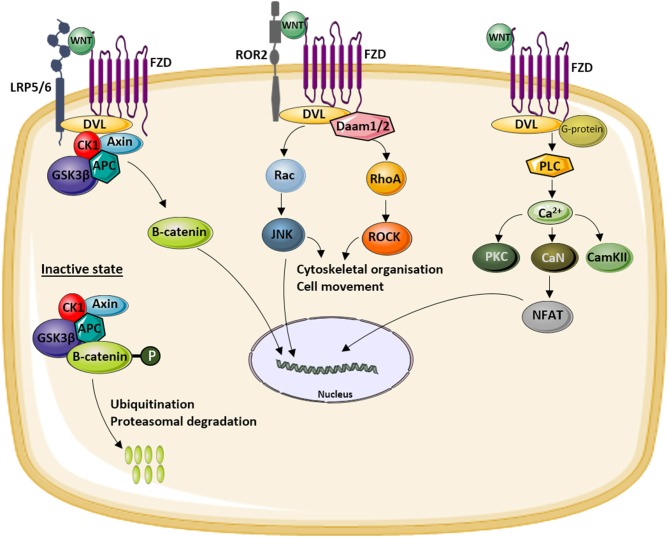
Overview of the different WNT signaling pathways. WNT ligands can activate the canonical or WNT/β-catenin signaling pathway (left) by binding to a receptor complex formed by low-density lipoprotein (LDL)-related receptor (LRP)5/6 and Frizzled (FZD). This activation results in the translocation of hypophosphorylated β-catenin to the nucleus. In the inactive state, β-catenin is degraded by the proteasome after phosphorylation by a glycogen synthase kinase 3β (GSK3β)-Axin-casein kinase 1 (CK1)–adenomatous polyposis coli (APC) complex and subsequent ubiquitination. The activation of the non-canonical WNT/planar cell polarity (PCP) pathway (middle) results in the activation of c-jun NH2-terminal kinase (JNK) *via* disheveled (DVL) and the Rac and Rho small GTPases. Activation of the non-canonical WNT/Ca^2+^ pathway (right) by binding of WNT to an FZD receptor results in intracellular Ca^2+^ release which activates a number of calcium-sensitive enzymes [protein kinase C (PKC), calcineurin (CaN), calmodulin-dependent protein kinase II (CamKII)]. More downstream nuclear factor of activated T cells (NF-AT) is activated and translocates to the nucleus to induce the expression of target genes.

The name WNT was introduced almost 30 years ago referring to the wingless-type mouse mammary tumor virus integration site (int-1) which was the first member from this gene family being described (1, 2). Meanwhile, three different pathways have been described, one canonical pathway also known as the WNT/β-catenin pathway and two non-canonical pathways, the WNT/Ca^2+^-dependent pathway and the WNT/planar cell polarity (PCP) pathway (Figure 1). All three are initiated by the binding of an extracellular WNT ligand to the N-terminal domain of one of the seven transmembrane-span FZD receptors. Depending on the pathway, also co-receptors are involved with the low-density lipoprotein (LDL)-related receptor (LRP)-5 and−6 being specific for canonical WNT signaling.

In mammalians, 19 different WNT ligands have been identified and 10 FZD receptors (3), already illustrating the involvement of this pathway in a broad range of cellular processes. All three pathways have a number of functions both during embryonic development and in adult life. These include cell fate specification, cell proliferation and migration, as well as body axis patterning. Furthermore, they are also important for cell functioning as well as processes of cell death. For some processes, only one of the three pathways is involved, but for others, evidence was generated indicating convergence of some of them (4).

At the beginning of this century, an additional role of WNT signaling was discovered following new gene identifications in some rare monogenic skeletal dysplasias. As explained in detail below, the study of conditions with either decreased or increased bone mass resulted in the identification of mutations in several genes involved in especially canonical WNT signaling (5). The most recent revision of the nosology and classification of genetic skeletal disorders (6) includes 461 different diseases. These disorders are interesting experiments of nature to gain insights into the regulatory mechanisms of bone formation, resorption, and homeostasis both during development and during adult life. In this review, we aim to discuss those skeletal disorders in which abnormal WNT signaling contributes to their pathogenesis. Furthermore, the implications of the novel insights toward more common bone disorders such as osteoporosis are highlighted.

## Extracellular Modulators

As previously mentioned, the WNT signaling pathway is activated by the binding of WNT ligands. Because of the broad functions of this pathway, additional regulation mechanisms are required to ensure proper timely and spatially functioning of the pathway. Extracellular modulators, including WNT inhibitors and activators, contribute to this complex regulation. Not surprisingly, mutations in various components of this pathway have been described in skeletal dysplasias.

### WNT Ligands

WNT ligands are secreted glycoproteins with a length of 350–400 amino acids. In humans, 19 different ligands have been identified, all containing 23–24 conserved cysteine residues (7, 8). A distinction between canonical (e.g., WNT1 and WNT3) and non-canonical (e.g., WNT5A) WNTs can be made, although overlap between the different pathways has been suggested. Various WNT ligands are associated with skeletal disorders, as described below.

#### WNT1

WNT1 is of major importance for the regulation of bone homeostasis, through binding with the co-receptor LRP5. Mutations in the *WNT1* gene are found in families with osteogenesis imperfecta (OI) type XV and early-onset osteoporosis (9–11).

OI is a hereditary connective tissue disorder, characterized by bone fragility, hearing loss, and dentinogenesis imperfecta. In the majority of patients, the disease is caused by heterozygous mutations in *COL1A1* and *COL1A2*, which encode the type I collagen pro α-chains. However, also recessive forms of OI do exist. One example is OI type XV caused by bi-allelic loss-of-function mutations in the *WNT1* gene (11–15). Moreover, the importance of the WNT signaling pathway in OI is emphasized by the observation of increased serum levels of Dickkopf1 (DKK1), an antagonist of the canonical WNT pathway, in OI patients. However, so far, no disease-causing OI mutations have been described in DKK1 (16).

Osteoporosis is a common skeletal disorder characterized by low bone mass, impaired bone quality, and increased fracture risk (14, 17). Whereas, most patients show symptoms later in life, early-onset osteoporosis can already present in adolescence. Some of this early-onset forms of osteoporosis are caused by heterozygous *WNT1* mutations (10, 12–15). These mutations result in disturbed bone remodeling and subsequent imbalance in bone homeostasis (13).

#### WNT3

WNT3 is critical for axis formation and limb growth in vertebrates (18, 19), and a homozygous nonsense mutation (p.Glu83^*^) in the *WNT3* gene has been reported in tetra-amelia syndrome type 1 (19). Tetra-amelia is an autosomal recessive disorder, characterized by the absence of all four limbs. It is extremely rare, with an incidence of 1.5–4 per 100,000 births (19–21).

#### WNT5A

Unlike the other WNT ligands discussed in this review, WNT5A belongs to the non-canonical WNT/PCP pathway. In order to exert its functions, such as promoting osteoblast differentiation, WNT5A binds to the cysteine-rich domain of receptor tyrosine kinase-like orphan receptor (ROR) 1/2 (22–24). Heterozygous loss-of-function mutations in the *WNT5A* gene are associated with autosomal dominant Robinow syndrome (RS) type 1 (ADRS1) (24–27). RS is characterized by skeletal abnormalities (short stature, brachydactyly, and mesomelic limb shortening predominantly of the upper limbs), genital hypoplasia, and typical facial dysmorphisms. The disorder is genetically and clinically heterogeneous, showing both dominant and recessive inheritance patterns. Additional phenotypic features of RS include hypertelorism, dental problems, and kidney abnormalities.

#### WNT6

Acro-pectoro-vertebral dysplasia (F-syndrome) is a rare skeletal disorder with a dominant inheritance pattern (28, 29). Symptoms include carpal and tarsal synostosis, malformations of the first and second fingers with frequent syndactyly, and spina bifida occulta (28–30). Although the molecular basis remains unknown, Thiele et al. (29) mapped the disease to a 6.5 cM region on chromosome 2q36, where the *WNT6* gene is located. As this gene is expressed during limb development, it is a promising candidate gene for harboring disease-causing mutations for F-syndrome (6, 29).

#### WNT7A

WNT7A forms a receptor complex with LRP6 and FZD5 in order to activate the canonical WNT signaling pathway (31, 32). It is expressed in the dorsal ectoderm and involved in the formation of the dorsoventral axis in limb development (32–34). Moreover, it also contributes to anteroposterior patterning by maintaining sonic hedgehog expression (32–34). Consistently, mutations in *WNT7A* cause limb malformations in Al-Awadi–Raas–Rothschild syndrome (AARRS) and Fuhrmann syndrome (FS) (31–37). Patients with AARRS display short upper limbs, severe malformations of the lower limbs, hypoplastic pelvis, anteriorly displaced genitalia, underdeveloped nails, and absence of uterus. The phenotype observed in FS is milder (e.g., solely ulnar deficiency and oligodactyly) since this is due to only a reduced function of the protein compared to complete loss-of-function mutations as seen in AARRS (32, 34–37). Although both are rare autosomal recessive disorders, different mutations contribute to the highly variable disease phenotypes (31, 34–36, 38, 39).

#### WNT10B

The WNT10B ligand is a key regulator for osteogenesis and is involved in all stages of limb development (24, 40–42). WNT10B plays an important role in the development of hands and feet, and bi-allelic mutations can cause split-hand/foot malformation type 6 (SHFM6). Generally, SHFMs are complex limb malformations that affect the central rays of hands and feet (42–44). From a clinical and genetic point of view, SHFM is highly heterogeneous. Both isolated and syndromic forms can be distinguished, as well as reduced penetrance has been reported (42–44). While most types of SHFM are autosomal dominantly inherited, SHFM6 follows an autosomal recessive pattern of inheritance (43, 44).

### WNT Inhibitors

#### SOST

Sclerostin, encoded by the *SOST* gene, is an osteocyte-derived secreted glycoprotein with a cysteine-knot motif (45). It acts as an extracellular antagonist of the canonical WNT signaling pathway by binding to the first β-propeller domain of LRP5/6 (46–49). Hence, sclerostin functions as a key regulator in bone metabolism and, particularly, bone formation (46, 50). Moreover, an interaction with the extracellular domain of LRP4 has been described, and studies demonstrated that this interaction is essential for normal sclerostin functioning (51, 52).

*SOST* was identified as the disease-causing gene for several high bone mass disorders, including sclerosteosis type 1. This rare skeletal dysplasia, caused by bi-allelic loss-of-function mutations in the *SOST* gene, is characterized by hyperostosis, tall stature, and syndactyly. The bone overgrowth affects predominantly the skull, mandible, and tubular bones in the limbs. Involvement of the skull base may cause compression of the cranial nerves and subsequent facial palsy, hearing loss, and blindness (50, 53–55). In 1998, Van Buchem disease, another autosomal recessive high-bone mass disorder, was mapped to the same chromosomal region as sclerosteosis (56). The molecular cause was a bi-allelic 52-kb deletion 35-kb downstream of *SOST* (23, 57). Since the deleted region contains a myocyte-enhancer factor 2C (Mef2c) binding site, imperative for *SOST* expression, the deletion results in reduced transcription of *SOST* (23). Although there is phenotypic overlap between sclerosteosis and Van Buchem disease, the phenotype of the latter is usually milder, as the patients have normal stature and do not exhibit syndactyly (54, 55). Another very rare sclerotic bone disorder is craniodiaphyseal dysplasia (CDD), inherited in an autosomal dominant manner. Clinical manifestations include sclerosis and hyperostosis, especially of the skull and facial bones, resulting in facial dysmorphism or “leontiasis ossea” (49, 58). Mutations causing CDD are located in the signal peptide of sclerostin and affect the secretion of sclerostin in a dominant negative manner.

#### GREM1

Gremlin has been characterized as a bone morphogenetic protein (BMP) inhibitor that regulates limb and kidney formation during development (59). However, WNT antagonistic activity has also been described since luciferase assays showed reduced stimulatory effects of WNT3A in the presence of gremlin (60, 61). In 2010, Dimitrov et al. (62) described a monoallelic 1.7 Mb duplication containing both the *GREM1* and the *FMN1* locus in a patient with Cenani–Lenz-like non-syndromic bilateral oligosyndactyly. *FMN1* contains a *cis*-regulatory region that is essential for the activation of gremlin in the limb bud. Hence, genomic rearrangements can lead to an altered function of gremlin and subsequent phenotypic abnormalities (39, 62–64). Interestingly, the phenotype of the patient is similar to that of the transgenic chick with overexpression of *Grem1* (65, 66). Furthermore, *GREM1* has been suggested to decrease the metastatic potential of osteosarcoma. Besides reduced gremlin levels in osteosarcoma cells, downregulation of *GREM1* was also associated with an increased degree of proliferation and angiogenesis (67).

#### SFRP4

Secreted FZD-related protein (sFRP) 4 is one of the five members of the sFRP family, known for antagonizing the WNT signaling pathway (68–70). sFRPs contain a C-terminal netrin-like domain (NTR) and an N-terminal cysteine-rich domain, sharing up to 50% sequence homology with the cysteine-rich domain of FZD receptors (24, 68, 70, 71). Consequently, sFRPs serve as decoy receptors for WNT ligands, thereby preventing activation of both canonical and non-canonical WNT signaling (24, 68–70). sFRP4 is a 346-amino acid protein, expressed in the majority of tissues (70). Regarding skeletal tissue, it contributes to maintaining normal osteoblast and osteoclast function during bone development and in the adult skeleton (68–70). A disrupted function of sFRP4 was discovered in Pyle disease, a recessive disorder affecting long bones with metaphyseal widening, cortex thinning, increased trabecular bone, decreased bone mineral density, and bone fragility (24, 72–74). Since 2016, various bi-allelic truncating mutations in *sFRP4* have been confirmed to cause Pyle disease (70, 72, 73). These nonsense mutations prohibit appropriate WNT and BMP signaling interaction, which is crucial to maintain appropriate stability of cortical bone (70, 71, 73).

### WNT Activators

#### Glypicans

Glypicans (GPCs), a subfamily of heparin sulfate proteoglycans, are linked to the cell surface *via* a glycosyl-phosphatidylinositol (GPI) anchor (75–77). The main function of the GPCs is regulating the activity of growth factors, such as hedgehogs and (non-)canonical WNTs (77–80). Six highly conserved GPCs have been identified in mammals, and pathogenic variants in *GPC4* and *GPC6* have been found in patients clinically diagnosed with skeletal dysplasia.

#### GPC4

GPC4 is located on chromosome Xq26 and is a regulator of the WNT/PCP pathway (79, 81). Only recently, hemizygous truncating and frameshift mutations were found in families with Keipert syndrome (82). These mutations are recognized as loss of function due to the subsequent loss of essential domains, such as the GPI anchor. Keipert syndrome is characterized by craniofacial and digital abnormalities, accompanied by learning difficulties and deafness (82, 83). Furthermore, there are indications that hemizygous pathological variants could result in an X-linked form of RS. White et al. (84) described a non-synonymous missense variant (p.Arg412Lys) in a male patient with an RS-like phenotype, including brachydactyly, mesomelia, and facial dysmorphisms.

#### GPC6

GPC6 is the smallest protein of the GPC family and is widely expressed in the growth plate during skeletal development (76, 77, 85). Genetic null mutations in *GPC6* prevent anchoring to the membrane, thereby disrupting the action of GPC6 in developmental processes (75–77). Such mutations were found in omodysplasia type 1 (OMOD1), an autosomal recessive skeletal dysplasia. Both nonsense mutations and genomic rearrangements have been described to contribute to the development of OMOD1 (76). This rare syndrome is characterized by cranial dysmorphisms, short stature of prenatal onset with severe shortening of the humeri (“omo” is the Greek term for humerus) and femora, and restricted mobility in elbows, hips, and knees. In addition, extra-skeletal manifestations, including congenital heart defects and cognitive delay, can also be observed (76, 77). There is also an autosomal dominant form of OMOD (OMOD2), which is milder with normal stature and is caused by heterozygous mutations in the *FZD2* gene (see below).

#### RSPO2

R-spondins (RSPO) are secreted proteins and activators of the canonical WNT signaling pathway (86–88). The WNT agonistic activity is a direct result of the formation of a ternary complex with leucine-rich repeat-containing G protein-coupled receptor 4, 5, or 6 (LGR4/5/6) and Zinc and ring finger 3 (Znrf3) or Ring finger protein 43 (Rnf43), as this complex prevents ubiquitination of the WNT receptors (86, 89–91). RSPOs are key regulators in bone development and bone homeostasis, partly mediated by RSPO2, which has high expression levels in the apical ectodermal ridge of limb buds, but also in the postnatal skeleton, lungs, and brain tissue (86). Similar to *WNT3* alterations (see above), an impaired RSPO2 function contributes to the development of tetra-amelia. However, patients affected with *RSPO2* mutations exhibit additional hypoplasia or aplasia of the lungs (91). Bi-allelic nonsense mutations, deletions, and frameshift mutations were described in families with tetra-amelia type 2 (91). Furthermore, a homozygous missense mutation (p.Arg69Cys) was reported in humerofemoral hypoplasia with radiotibial ray deficiency, albeit in only one consanguineous family. The affected family members showed severe dysostosis with malformation of all four limbs, absence of tibiae, and hypoplasia of the pelvis (91).

## Receptors and Co-Receptors

Both the canonical and non-canonical WNT signaling pathways are activated by the binding of WNT ligands to the FZD receptors alone or in combination with specific co-receptors. Identification of single disease mutations in LRP5, LRP6, and ROR2 highlights the importance of these co-receptors in the regulation of canonical and non-canonical WNT signaling during skeletal development.

### FZD2

FZD2 is a member of the FZD receptor family that includes nine G protein-coupled receptors. These FZD receptors interact with disheveled (DVL) upon activation by one of the WNT ligands which results in the initiation of one of the different WNT pathways. In 2015, Saal et al. (92) demonstrated that mutations in *FZD2* can cause autosomal dominant OMOD, also referred to as OMOD2. OMOD2 is a rare skeletal dysplasia which is marked by severe rhizomelic shortening of the upper limbs, mild facial dysmorphism (frontal bossing, depressed nasal bridge, and short nose), and genital hypoplasia (92). Clinically, OMOD2 can be distinguished from OMOD1 (recessive form—discussed previously) by the presence of short first metacarpals and normal stature (93).

As mentioned, heterozygous mutations in *FZD2* can cause OMOD2. To date, one missense mutation (p.Gly434Val) and two nonsense mutations involving adjacent amino acids (p.Ser547^*^ and p.Trp548^*^) have been reported (92–95). The nonsense mutations are located in the intracellular domain of FZD2, more precisely in the conserved DVL binding motif (KTxxW). Subsequent functional studies have demonstrated that in the presence of p.Trp548^*^ mutant FZD2, the localization of DVL at the membrane and the activation of the canonical WNT signaling pathway upon WNT treatment are lost (92). The effect of the identified mutations on the non-canonical WNT signaling pathways was not investigated. Several of the clinical features that are reported in patients with OMOD2 are also present in patients with RS (described previously). Therefore, it is likely that the causal genes for these disorders are involved in the same pathway(s). As it is shown that WNT5A/ROR2 interaction can induce WNT/PCP signaling (see below), it is likely that FZD2 is also involved in this pathway.

### ROR2

ROR2 is a member of the ROR kinase family of orphan receptors with tyrosine kinase activity which is involved in the WNT signaling pathway as co-receptor of the non-canonical PCP pathway. The extracellular domain of ROR2 which is important for protein–protein interactions contains several conserved domains such as an immunoglobin-like domain, an FZD-like cysteine-rich domain (CRD), and a Kringle domain (KD). The intracellular region contains a tyrosine kinase (TK) domain followed by serine/threonine-rich and proline-rich structures. Mutations in *ROR2* can cause either autosomal dominant brachydactyly type B1 (BDB1) or autosomal recessive RS type 1 (ARRS1) (96, 97).

ARRS1 is characterized by short stature, mesomelic limb shortening, genital hypoplasia, and typical facial dysmorphisms (including hypertelorism). In addition, patients with ARRS1 may also have vertebral segmentation defects such as hemivertebrae and rib fusions (98). Since the identification of *ROR2* as a disease-causing gene for ARRS1, more than 25 different mutations have been identified (99). These mutations include missense, nonsense, and frameshift mutations located in both the intra- and extracellular regions of the protein (96, 98–101). Based on the phenotypic overlap between ARRS1 and *Ror2* knockout mouse, it was suggested that the mutations result in loss of function of ROR2 due to decreased protein stability and intracellular entrapment of the mutant protein (98, 101). ROR2 serves as a co-receptor for WNT5A-induced non-canonical WNT signaling of which WNT/PCP signaling is shown to be most important for limb development. Loss of function of ROR2 will most likely result in decreased WNT/PCP signaling and limb deformities (102).

BDB1 is the most severe form of brachydactyly and is one of the first described types of heritable brachydactyly. It is characterized by hypoplasia and/or aplasia of the distal phalanges and nails in hand and feet resulting in an amputation-like phenotype (101, 102). The BDB1 causing mutations in *ROR2* are all nonsense or frameshift mutations that are located in the intracellular region of the protein and co-localize in two domains, either immediately N-terminal or C-terminal of the TK domain (101). Genotype–phenotype correlation demonstrated that the latter group of mutations results in a more severe phenotype (103). Since parents of ARRS1 patients do not show brachydactyly, it is suggested that BDB1 causing mutations are gain-of-function mutations (96).

### LRP Receptors

The LRPs are a group of evolutionary conserved receptors regulating a wide range of cellular processes. In order to regulate these processes, LRPs can modulate a variety of pathways, including canonical WNT signaling activity (104). The extracellular region of the majority of the LRP receptors contains at least one ligand-binding domain composed of cysteine-rich ligand-binding-type repeats and one epidermal growth factor (EGF)-precursor homology domain composed of EGF repeats and a YWTD/β-propeller domain. Most LRP receptors have in the intracellular region at least one NPxY-(endocytosis) motif. However, LRP5/6 are lacking this motif (105). Mutations in three members of the LRP family, namely, LRP4, LRP5, and LRP6, are reported to result in rare skeletal disorders (51, 106, 107).

### LRP5

Almost 20 years ago, genetic research highlighted the importance of LRP5 in the regulation of bone formation with the identification of disease-causing mutations in patients with abnormal low or high bone mass. Subsequent studies demonstrated that LRP5, as a co-receptor of the canonical WNT signaling pathway, regulates osteoblast proliferation and differentiation as well as osteocyte apoptosis (108). More specifically, homozygous loss-of-function mutations in LRP5 can cause the osteoporosis-pseudoglioma (OPPG) syndrome which is characterized by reduced bone mass, increased bone fragility, and severely reduced visual acuity (109). So far, over 70 different mutations in *LRP5* have been reported to cause OPPG, and these mutations are spread throughout the gene. Mutations affecting splicing or introducing premature stop codons result in the most severe phenotypes (110). Furthermore, reduced bone mass is also reported in heterozygous mutation carriers (111). Besides the severe OPPG phenotype, it is reported that heterozygous OPPG loss-of-function mutations in *LRP5* can cause juvenile-onset osteoporosis without an ocular phenotype in children (112).

As mentioned, mutations in *LRP5* can also result in a skeletal phenotype with increased bone mass (106, 113, 114). When consulting OMIM or the nosology of skeletal disorders (6), mutations in *LRP5* are identified in patients diagnosed with high bone mass (HBM) phenotype, autosomal dominant osteopetrosis type 1, autosomal dominant Van Buchem disease, Worth disease, endosteal hyperostosis, or osteosclerosis. Although these rare disorders have a different name, they represent the same disorder which is characterized by increased bone mass especially affecting the skull and tubular bones and a reduced fracture risk (106). Due to the increased bone mass of the skull, headaches and cranial nerve entrapment are commonly reported in these patients (115). Furthermore, these disorders are all caused by heterozygous gain-of-function mutations in *LRP5*. All mutations are located in the first β-propeller domain of the protein, and functional studies demonstrated that they disrupt the binding of canonical WNT signaling inhibitors sclerostin and DKK1 with the co-receptor. Although mutations in DKK1 are not reported in patients with monogenic skeletal disorders, different studies have shown that DKK1 is an important regulator of the WNT signaling pathway and bone mass *via* its interaction with LRP5 (116). As a result, mutations disrupting the binding of LRP5 with sclerostin and DKK1 result in increased canonical WNT signaling activity which consequently leads to increased bone formation (116–118).

### LRP6

LRP6 is the closest homolog of LRP5, and functional studies demonstrated that LRP6, similar to LRP5, is a co-receptor of the canonical WNT signaling pathway (104). However, mutations in *LRP6* are less common compared to *LRP5*, most likely due to a more important role for LRP6 during embryonic development. However, recently, heterozygous mutations in the first β-propeller domain of LRP6 were identified in two families with HBM phenotype. The amino acids mutated in LRP6 are homologs to known LRP5 mutations (107). Although no functional studies were performed, it is highly likely that the mutations, similar to those reported for LRP5, disturb the binding of sclerostin and DKK1 and consequently result in increased WNT signaling activity and increased bone mass. The skeletal phenotype of LRP6 mutant patients was similar to the phenotype reported in patients with LRP5 mutations. However, in the small group of patients with HBM mutations in LRP6, no cranial nerve entrapment was reported which is commonly found in LRP5 HBM mutant patients. In addition, in patients with HBM mutations in LRP6, absence of the adult maxillary lateral incisors was reported. Since the latter is never reported in LRP5 HBM mutant patients, this finding can maybe be used to distinguish between both HBM types (107). Besides the high bone mass phenotype, heterozygous loss-of-function mutations in *LRP6* are previously shown to cause coronary artery disease and tooth agenesis (119, 120). Remarkably, in the family with coronary artery disease due to a mutation in *LRP6* described by Mani et al. (119), osteoporosis was also reported. Based on the role of LRP6 in WNT signaling, it was suggested that the observed osteoporosis phenotype was also caused by the mutation in *LRP6*.

### LRP4

Another LRP receptor which is implicated in the regulation of bone formation is LRP4. In contrast to LRP5/6, LRP4 is involved in the inhibition of the canonical WNT signaling pathway through its interaction with sclerostin. The identification of disease-causing *LRP4* mutations in syndromic disorders demonstrated that LRP4 is not only important in the regulation of bone mass but also regulates limb and kidney development among others (51, 121, 122). Mutations in *LRP4* can cause sclerosteosis type 2, Cenani–Lenz syndrome (CLS), isolated syndactyly, and congenital myasthenia gravis depending on type and location of the mutations (51, 122–124). As myasthenia gravis patients have no skeletal phenotype and the mutations do not affect WNT signaling activity (125), it is not further discussed in this review.

The clinical phenotype of sclerosteosis type 2 is highly similar to the sclerosteosis type 1 phenotype previously described. Sclerosteosis type 2 causing mutations in *LRP4* are located in the third β-propeller domain and result in decreased binding of sclerostin (51, 124). Due to the impaired LRP4–sclerostin binding, serum levels of sclerostin are elevated in these patients and the activity of the canonical WNT signaling pathway is increased in osteoblasts, leading to increased bone formation (51, 124).

As mentioned, bi-allelic mutations in *LRP4* can also cause CLS. CLS is marked by syndactyly, synostosis, and renal abnormalities, similar to what has been discussed above for patients with a mutation in *GREM1* that interacts with LRP4. CLS-causing mutations are spread throughout LRP4, except for the third β-propeller domain (122, 123). Functional studies indicated CLS-causing mutations result in a decreased expression of the receptor at the cell membrane. This indicates that CLS-causing mutations have an effect not only on the inhibition of the canonical WNT signaling pathway by sclerostin but also on the regulation of other pathways such as MUSK-agrin signaling. In addition, in an individual with isolated syndactyly, compound heterozygous missense mutations within the fourth β-propeller domain have been described (126). *In vitro* studies demonstrated that the presence of the mutations results in decreased inhibition of the WNT signaling activity by LRP4 similar as reported for CLS mutations. Therefore, it is suggested that these patients might suffer from a mild type of CLS.

## Intracellular Regulators of WNT Signaling

Activation of the different FZD receptors and co-receptors results intracellularly in a complex signaling cascade which ultimately leads to transcription of target genes and regulation of a wide range of cellular processes. There are many proteins involved in this intracellular signaling; however, only a few underlie the development of rare inherited skeletal dysplasias.

### Disheveled

The disheveled protein family consists of three members (DVL1-3) which all act downstream of FZD receptors in both the canonical and non-canonical WNT signaling pathway. The DVL proteins share highly conserved regions, namely, the amino-terminal DIX (N-terminus) domain, a PDZ (central) domain, a carboxyl-terminal DEP (C-terminus) domain, and two regions with positively charged amino acid residues (127). These conserved regions are important to mediate protein–protein interactions and define the further propagation of the signal through either the canonical or non-canonical pathway. Furthermore, DVL can move from the cytoplasm to the nucleus, and this is also required for proper propagation of the WNT signaling. To ensure the movement of DVL to the nucleus, DVL proteins contain a nuclear export sequence (NES) and nuclear localization sequence (NLS) (128).

DVL proteins are highly important for normal functioning of the different WNT signaling pathways. Genetic defects can consequently affect the skeleton. Heterozygous mutations in both *DVL1* and *DVL3* have been identified in individuals with autosomal dominant RS (ADRS) types 2 and 3, respectively (129–131). As mentioned, ADRS is marked by skeletal abnormalities, genital abnormalities, and dysmorphic facial features (132).

### DVL1

In 2015, two independent research groups reported the identification of *de novo* frameshift mutations in exon 14 of *DVL1* in patients with ADRS without mutations in *WNT5A* (129, 130). In total, nine different frameshift mutations, all located in the penultimate exon of *DVL1*, have been reported so far. All mutations result in the same premature stop codon in the last exon and escape nonsense-mediated mRNA decay. The mutations are located downstream of the abovementioned conserved domains, and all mutations result in a novel shared, highly basic C-terminal tail sequence that most likely exerts novel signaling functions (129–131). More in detail, *in vitro* luciferase reporter assays demonstrated that co-transfection of wild-type and mutant DVL1, representing the heterozygous mutations, results in significantly increased canonical WNT signaling activity compared to the effect of wild-type DVL1 alone. More interestingly, several patients with mutations in *DVL1* show increased bone mass which is not seen in the other autosomal dominant or recessive forms of RS (129). Most likely, the increased bone mass in ADRS2 patients is caused by the effect of *DVL1* mutations on canonical WNT signaling. In addition, ADRS2 patients often have macrocephaly and normal stature (>10% percentile) (129, 130).

### DVL3

In a subsequent study, it was shown that not only mutations in *DVL1* but also *DVL3* can cause ADRS (131). Similar as for DVL1, the mutations result in a frameshift to the−1-reading frame and a shared premature stop codon in the last exon. The DVL3 mutants are shown to escape nonsense-mediated mRNA decay. All identified mutations have 83 C-terminal amino acids in common (131, 133). The functional effect of this C-terminal tail on regulation of canonical and/or non-canonical WNT signaling pathway is still unknown. Patients with mutations in *DVL3* (ADRS3) can be distinguished from ADRS2 patients based on the presence of short stature and macrocephaly. Furthermore, congenital heart defects and cleft lip and/or cleft palate were reported in the majority of the patients (131).

### NXN

Nucleoredoxin (NXN) is an oxidative stress response protein which is highly expressed during murine limb bud development and can directly bind DVL *via* the PDZ domain. By binding to DVL, NXN can inhibit DVL signaling activity and consequently different WNT signaling pathways (134–136). It is suggested that the interaction of NXN with DVL is a key regulatory mechanism to maintain spatial and temporal balance between canonical and non-canonical WNT signaling during development (84). Recently, *NXN* was identified as the causative gene for autosomal recessive RS type 2 (ARRS2). In two families without mutations in the known causative gene *ROR2*, White et al. (84) identified bi-allelic variants which segregated with the ARRS2 phenotype consisting of short stature, mesomelic shortening, genital hypoplasia, and typical facial dysmorphisms. Mice lacking Nxn also show craniofacial defects, suggesting that the mutations reported in the patients are loss-of-function mutations (136, 137).

### RAC3

RAC3 is a member of the Rac subfamily of the Rho family of GTPases. Upon activation by WNT ligands, RAC3 can interact with DVL to activate downstream signaling including c-jun NH2-terminal kinase (JNK)/c-jun phosphorylation which is necessary for cytoskeletal organization. White et al. (84) reported in 2018 the identification of a rare variant in RAC3 in a patient with a Robinow-like phenotype including facial dysmorphism and developmental delay, seizures, an abnormal electroencephalogram, and a thin corpus callosum. Based on the role of RAC3 in the WNT/PCP pathway (**Figure 3**), they suggest that the identified *de novo* variant can cause the Robinow-like phenotype in the patient (84). However, additional functional studies or identification of additional affected individuals with possible pathogenic variants is necessary. In a more recent study, Costain et al. (138) also identified heterozygous missense variants in *RAC3* in patients with neurodevelopmental disorder with structural brain anomalies and dysmorphic facial features. Since there is some overlap between the clinical phenotype of the patients described in both reports, most likely the patients described by both research groups suffer from the same disease.

### APC2

*Adenomatous polyposis coli* (*APC*) is a tumor suppressor gene that can regulate canonical WNT signaling as a part of the multiprotein destruction complex that targets β-catenin for phosphorylation and degradation (139). APC2 or APC-like protein is a homolog of APC which is mostly expressed in the postmitotic neurons during development (140). Both proteins share a 20-amino acid repeat motif that is capable of binding β-catenin. However, in addition to this shared motif, APC also contains a second motif which is lacking in APC2 and can bind β-catenin with a higher affinity. Nevertheless, APC2 is still capable of depleting intracellular β-catenin, although less efficient than APC (141).

Homozygous loss-of-function mutations in *APC2* have recently been described in patients with a Sotos-like phenotype (142). Sotos syndrome is an overgrowth syndrome, also known as cerebral gigantism. Individuals with APC2 mutations resemble patients with Sotos syndrome because they show intellectual disability and relative macrocephaly with a long face and prominent chin (142). The role of the canonical WNT signaling pathway in the development of this disorder is not yet investigated.

### AMER1

APC Membrane Recruitment Protein 1 (AMER1), also known as FAM123B or Wilms tumor on the X chromosome (WTX), is an intracellular inhibitor of the canonical WNT signaling pathway. AMER1 can interact with the AXIN/APC/GSK3β/β-catenin multiprotein complex and enhances the ubiquitination and degradation of β-catenin (143, 144). *AMER1* is located on the X-chromosome, and loss-of-function mutations or gene deletions can cause the X-linked dominant disorder osteopathia striata with cranial sclerosis (OSCS) (145). Radiographs of patients with OSCS show cranial sclerosis and dense linear striations in the submetaphyseal regions of the long bones and pelvis. Besides these radiographic signs, patients with OSCS often also present with macrocephaly, broad nasal bridge, frontal bossing, ocular hypertelorism, hearing loss, and palate abnormalities. Less common symptoms of OSCS are cardiac malformations and cognitive defects. In males, OSCS can be lethal due to severe heart defects and/or gastrointestinal malformations (143, 145, 146).

### PORCN

Porcupine O-acyltransferase (PORCN) is a member of the evolutionarily conserved porcupine (PORC) gene family of endoplasmic reticulum transmembrane proteins that are involved in the processing and secretion of WNT ligands. *PORCN* is located on the X-chromosome, and heterozygous loss-of-function mutations can cause focal dermal hypoplasia (FDH) also known as Goltz syndrome in female patients. Goltz syndrome caused by mutations in *PORCN* is rarely reported in males which leads to the suspicion that non-mosaic Goltz syndrome is embryonically lethal for males (147, 148). Goltz syndrome is a multisystem syndrome that is characterized by dermal abnormalities including focal dermal hypoplasia among others, ophthalmologic features, facial anomalies, and skeletal abnormalities. The latter are reported in ~80% of the patients and include syndactyly or polydactyly, osteopathic striae, hypoplasia or absence of digits, scoliosis, and/or facial asymmetry (148, 149).

## Discussion

**Figure 2:**
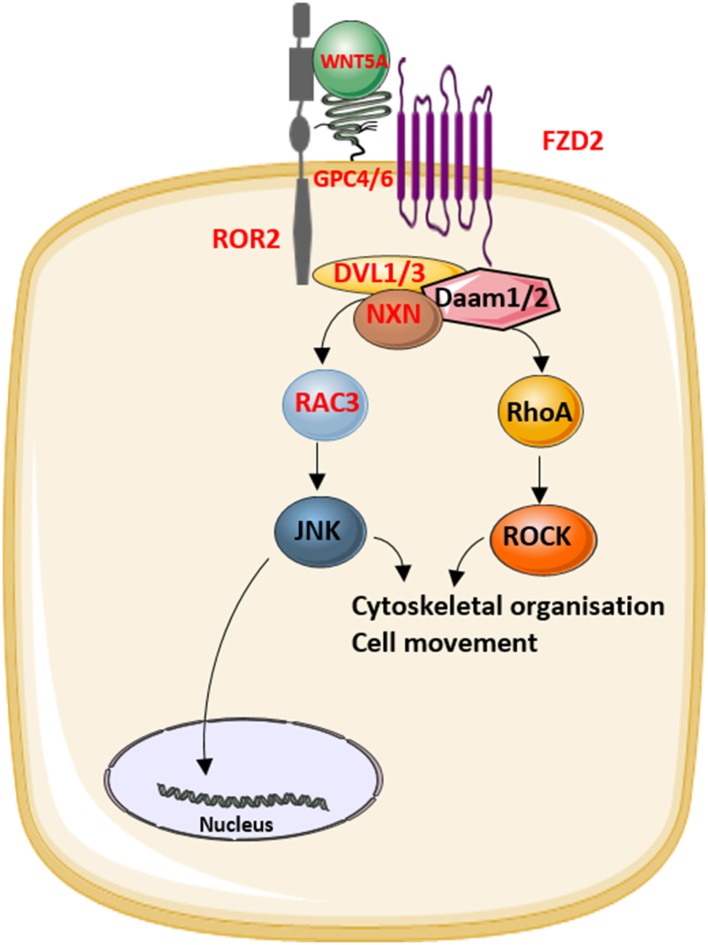
The role of the proteins that underlie Robinow syndrome or omodysplasia (indicated in red) in the WNT/planar cell polarity (PCP) pathway.

**Figure 3:**
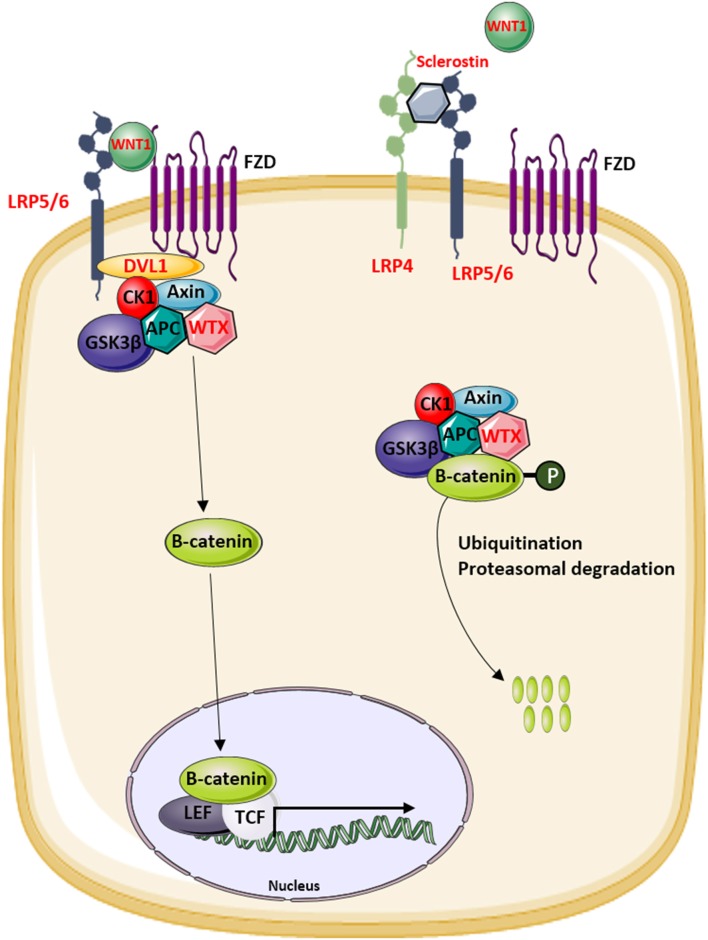
Overview of the modulators of the canonical WNT signaling pathway that are involved in the regulation of bone mass in human disease. Proteins indicated in red are mutated in patients with increased or decreased bone mass.

The identification of novel disease-causing genes for rare skeletal dysplasias accelerated significantly in the last decades, initially by positional cloning efforts and more recently by the availability of next-generation sequencing technology. This resulted in the identification of the disease-causing gene for 92% of the skeletal disorders (6). The increased knowledge on monogenic diseases resulted in a better understanding of the pathological mechanisms and highlighted which pathways regulate specific cellular processes. This information is also relevant for understanding more common multifactorial diseases. Furthermore, it has been shown that therapeutic targets which are based on genetic evidence from Mendelian traits as well as genome-wide association studies (GWASs) are more likely to be successful in clinical studies for multifactorial diseases (150). Here, we focused on skeletal dysplasias caused by mutations in genes that encode proteins that are directly involved in one of the WNT signaling pathways. As shown in Table 1, mutations in these genes can result in a variety of skeletal dysplasias, each with specific clinical features. The broad spectrum of clinical observations reflect the cellular and spatial functions of WNT signaling, some of them associated with embryonal development, others with bone mass and homeostasis in adult life. For example, the clinical features of RS and OMOD are similar which led to the hypothesis that all causative genes are involved in the WNT/PCP pathway which is previously shown to be important during limb development (Figure 2) (102). On the other hand, the influence of canonical WNT signaling on bone mass was highlighted by unraveling the underlying pathogenic mechanisms of disorders with a progressively increasing bone mass such as sclerosteosis, Van Buchem disease, and high bone mass phenotypes (osteosclerosis) (51, 53, 57, 107, 113). The genes causing these disorders, *SOST, LRP4, LRP5*, and *LRP6*, are all involved in the canonical WNT signaling pathway (Figure 3), and all mutations reported result in an increased canonical WNT signaling (Table 1). In addition to the conditions with increased bone mass mentioned, mutations in *WNT1*, a ligand inducing canonical WNT signaling, and *LRP5* can also result in decreased canonical WNT signaling activity and consequently decreased bone mass (Table 1; Figure 3) (12–15, 109). All these data show that the canonical WNT signaling pathway is an important regulator of bone mass as is also corroborated by the observation in GWASs that many of these genes harbor genetic polymorphisms associated with bone mineral density or fractures (151, 152, 154) (Table 1). This is in line with a general observation that there is an enrichment in genes for Mendelian disorders near regions associated in GWASs with a similar phenotype (157).

**Table 1:** Overview of the main skeletal dysplasias caused by mutations in genes involved in WNT signaling.

**Gene**	**Phenotype**	**OMIM**	**Skeletal**	**Other symptoms**	**LOF/GOF**	**Genetic associations**
**EXTRACELLULAR MODULATORS**						
*WNT1*	Osteogenesis imperfecta type XV (AR)	615220	Recurrent bone fractures Bone deformity Short stature Low bone mass	Blue sclerae (not all patients) Brain malformations (not all patients) Hearing loss Dentinogenesis imperfecta	LOF	BMD^a, b^
	Early-onset osteoporosis (AD)	615221	Recurrent bone fractures Low bone mass Impaired bone quality (trabecular and cortical)		LOF	
*WNT3*	Tetra-amelia syndrome type 1 (AR)	273395	Absence of all limbs Pelvis hypoplasia	Cleft lip/palate Pulmonary abnormalities Urogenital defects Kidney/spleen/adrenal glands hypoplasia	LOF	/
*WNT5A*	Robinow syndrome type 1 (AD)	180700	Short stature Mesomelic limb shortening Hypertelorism Mandibular hypoplasia Dental problems	Genital hypoplasia Kidney abnormalities	LOF	/
*WNT6*	Acro-pectoro-vertebral dysplasia (F-syndrome, AD)	102510	Carpal/tarsal synostoses Skeletal malformations Syndactyly/polydactyly	Craniofacial anomalies Spina bifida occulta	LOF	/
*WNT7A*	Fuhrmann syndrome (AR)	228930	Limb shortening Fibular/ulnar hypoplasia Oligosyndactyly		Partial LOF	/
	Al-Awadi–Raas–Rothschild syndrome (AR)	276820	Limb shortening Absence of ulna/fibula Oligosyndactyly Pelvis hypoplasia	Kidney agenesis Genital hypoplasia (females: absence of uterus, anteriorly displaced genitalia) Hypoplasia/absence of nails	LOF	
*WNT10B*	Split-hand/foot malformation type 6 (AR)	225300	Malformations of hands/feet		LOF	**B**MD^c^
*SOST*	Sclerosteosis type 1 (AR)	269500	Progressive skeletal overgrowth (tubular bones, skull, mandible) Tall stature Syndactyly	Cranial nerve compression due to increased bone mass of the skull	LOF	BMD^a, b^/fractures^a, d^
	Van Buchem disease (AR)	239100	Increased thickness of bones (tubular bones, skull, mandible)	Cranial nerve compression due to increased bone mass of the skull	Partial LOF	
	Craniodiaphyseal dysplasia (AD)	122860	Sclerosis Hyperostosis (skull/facial bones) Facial distortion (= leontiasis ossea)	Severe neurologic impairment	LOF (dominant negative)	
*Grem1*	Cenani–Lenz-like non-syndromic oligosyndactyly (SP/AD)	NA	Bilateral oligosyndactyly		LOF	/
*sFRP4*	Pyle disease (AR)	265900	Metaphyseal widening (long bones) Cortex thinning Increased trabecular bone mass Decreased bone mineral density Fractures Genu valgum Dental abnormalities		LOF	BMD^e^/fractures^a^
*GPC4*	Keipert syndrome (X-linked)	301026	Craniofacial abnormalities Digits abnormalities	Learning difficulties Deafness	LOF	/
	Robinow-like phenotype (X-linked)		Brachydactyly Mesomelia Facial dysmorphism			
*GPC6*	Omodysplasia type 1 (AR)	258315	Short stature Severe limb malformations (shortening of humeri/femora) Restricted mobility in elbows/hip/knees Craniofacial dysmorphism	Cryptorchidism Hernias Congenital heart defects Cognitive delay	LOF	BMD^a, b^
*RSPO2*	Tetra-amelia syndrome type 2 (AR)	618021	Absence of all limbs Pelvis hypoplasia	Lung hypo/aplasia Cleft lip/palate Dysmorphic features	LOF	BMD^a^
	Humerofemoral dysplasia (AR)	618022	Severe dysostosis Malformation of all limbs Absence of tibiae Femoral deficiency (not all patients) Absence of digits (preaxial side) Pelvis hypoplasia		LOF	
**(CO-)RECEPTORS**						
*FZD2*	Omodysplasia type 2 (AD)	164745	Craniofacial dysmorphism Limb shortening	Genital hypoplasia	LOF	/
*ROR2*	Robinow syndrome type 1 (AR)	268310	Short stature Limb shortening Facial dysmorphisms	Genital hypoplasia	LOF	BMD^f^
	Brachydactyly type B1 (AD)	113000	Hypoplastic/aplastic distal phalanges and nails in hands and feet Hypoplastic middle phalanges		GOF	
*LRP5*	Osteoporosis-pseudoglioma syndrome (AR)	259770	Reduced bone mass and strength	Blindness due to abnormal blood vessel development in the eye	LOF	BMD^a, b^/fractures^d^
	(Juvenile) osteoporosis (AD)		Reduced bone mass and strength		LOF	
	Endosteal hyperostosis (AD)	144750	Cortical thickening of the long bones Generalized sclerosis Increased thickness of the skull Increased bone strength Torus palatinus (some patients)	Cranial nerve compression due to increased bone mass of the skull	GOF	
	Osteopetrosis type 1 (AD)	607634				
	Van Buchem disease type 2 (AD)	607636				
	High bone mass phenotype (AD)	601884				
*LRP6*	High bone mass phenotype (AD)		Cortical thickening of the long bones Generalized sclerosis Increased thickness of the skull Increased bone strength	absence of the adult maxillary lateral incisors	GOF	BMD^a, b^
*LRP4*	Sclerosteosis type 2 (AR)	614305	Progressive skeletal overgrowth Cortical thickening of the tubular bones and skull Facial asymmetry Syndactyly	Cranial nerve compression due to increased bone mass of the skull	Partial LOF	BMD^a, b^
	Cenani–Lenz syndrome (AR)	212780	Distal bone malformations Syndactyly Mild facial dysmorphism	Kidney anomalies	LOF	
**INTRACELLULAR REGULATORS**						
*DVL1*	Robinow syndrome type 2 (AD)	616331	Limb shortening Facial dysmorphisms Osteosclerosis Short stature (rare)	Genital hypoplasia	LOF/GOF*	/
*DVL3*	Robinow syndrome type 3 (AD)	616894	Limb shortening Facial dysmorphisms Short stature Macrocephaly (not all patients)	Genital hypoplasia	LOF	/
*NXN*	Robinow syndrome type 2 (AR)	618529	Limb shortening Facial dysmorphisms Short stature Macrocephaly Brachydactyly	Congenital anomalies (omphalocele, ventral hernia, and cardiac anomalies)	LOF	BMD^a, b^
*RAC3*	Robinow-like phenotype		Facial dysmorphism	Developmental delay Seizures Abnormal electroencephalogram Thin corpus callosum	LOF	/
*APC2*	Sotos syndrome type 3 (AR)	617169	Macrocephaly Long face Prominent chin and nose	Severe receptive and expressive language disorder, learning disabilities, and hyperactive behavior	?	/
*AMER1*	Osteopathia striata with cranial sclerosis (X-linked)	300373	Sclerosis of the long bones and skull Longitudinal striations in the long bones, pelvis, and scapulae Macrocephaly	Cardiac, intestinal, and genitourinary malformations (males) Cleft palate	LOF	/
*PORCN*	Goltz syndrome (X-linked)	305600	Syndactyly, ectrodactyly, polydactyly Osteopathic striae Hypoplasia or absence of digits Scoliosis Facial asymmetry	Dermal abnormalities (focal dermal hypoplasia, subepidermal subcutaneous fat deposits, …) Ocular, urinary, gastrointestinal, cardiovascular, neurologic, and oral abnormalities	LOF	/

*Combined expression of WT and mutant DVL1 results in increased canonical WNT signaling; however, the effect of DVL1 on the WNT/PCP pathway is most likely LOF based on the LOF mutations identified in ROR2 and WNT5A.

AD, autosomal dominant; AR, autosomal recessive; BMD, bone mineral density; DVL, disheveled; GOF, gain of function; LOF, loss of function; PCP, planar cell polarity; WT, wild type.

*^a^Morris et al. (151); ^b^Kichaev et al. (152); ^c^Van Camp et al. (153); ^d^Trajanoska et al. (154); ^e^Wang et al. (155); ^f^Mullin et al. (156)*.

The evidence for an important role for WNT signaling in regulating bone mass makes it an obvious candidate for drug development for the treatment of osteoporosis. The incidental finding that lithium chloride (LiCl), a first-line treatment for bipolar disorders, increases canonical WNT signaling activity and increases bone formation already confirms the therapeutic potential of the pathway (158, 159). But at the same time, phenotypic features of single mutation diseases can give insights into possible adverse effects of interfering with the pathway. For example, a rare nonsense mutation (c.376C>T) was identified in the *LGR4* gene, which encodes a receptor for RSPO1–4, activators of WNT signaling. The mutation is specific for the Icelandic population, as described by Styrkarsdottir et al. (160), and shows strong association with osteoporotic fractures and low bone mineral density (BMD). But at the same time, the variant is associated with an increased risk of squamous cell carcinoma of the skin and biliary tract cancer (160). Also for other types of cancer, abnormal WNT signaling has been reported. These cancers can be caused by single mutations (germline or mosaic) in, for example, *APC* or *WTX*, but also common genetic variation in *WNT3, DVL1*, and *NXN* is previously associated with increased cancer risk (161–166). However, increased cancer risk is not reported for any of the rare skeletal dysplasias described in this review.

Currently, the treatment of osteoporosis is mainly based on the use of antiresorptive agents, such as bisphosphonates, without the capacity to rebuild the lost bone. Consequently, there is an urgent need for more specific, anabolic therapies for osteoporosis, making canonical WNT signaling of major interest. The pharmaceutical industry focused on sclerostin as a therapeutic target based on the expression of sclerostin being almost exclusive in bone tissue, the progressive increase in bone mass in adults with sclerosteosis and Van Buchem disease and on the absence of non-skeletal clinical features in these patients (167). Clinical trials demonstrated that treatment with romosozumab (Evenity™, Amgen/UCB), a monoclonal antibody for sclerostin, results in increased bone mass and decreased fracture risk (168, 169). However, a phase III clinical trial demonstrated that although romosozumab is clearly beneficial for bone health, treatment was also associated with slightly increased incidence of cardiovascular events (169). Increased incidence of cardiovascular events was observed in men treated with romosozumab compared to placebo (170) and in postmenopausal women treated with romosozumab compared to women treated with alendronate (169). When compared to postmenopausal women treated with placebo, romosozumab-treated individuals have a similar incidence of cardiovascular events (168). In addition, in patients pretreated with alendronate, the incidence of cardiovascular events was lower (171). Furthermore, in postmenopausal women who were treated with alendronate after romosozumab treatment, the difference in cardiovascular events remained stable compared to patients treated with alendronate alone (169). Therefore, additional studies are needed to investigate whether combined treatment of alendronate and romosozumab can reduce the cardiovascular events. Clinical data from sclerosteosis and Van Buchem patients who lack sclerostin do not report an increased incidence of cardiovascular events in these patients (172) which suggests that targeting sclerostin does not cause cardiovascular disease. However, for now, the use of romosozumab is only approved for the treatment of severe osteoporosis in postmenopausal women but is contraindicated in patients with an increased cardiovascular risk. Therefore, the identification of new therapeutic targets potentially interfering with WNT signaling remains imperative.

In conclusion, mutations in the WNT signaling pathway can lead to a broad range of skeletal dysplasias. Especially those characterized by an increased bone mass have been instrumental in highlighting the role of WNT signaling in bone formation and homeostasis with therapeutic applications for the treatment of osteoporosis.

## Author Contributions

All authors listed have made a substantial, direct and intellectual contribution to the work, and approved it for publication.

### Conflict of Interest

The authors declare that the research was conducted in the absence of any commercial or financial relationships that could be construed as a potential conflict of interest.
